# Supplementary material for: Patterns of Intron Gain and Loss in Fungi
Source: PLoS Biol. 2004 Nov 30;2(12):e422. doi: 10.1371/journal.pbio.0020422 (PMC532390; doi:10.1371/journal.pbio.0020422)
Supplement: Table S1 — Also available at http://genes.mit.edu/NielsenEtAl/. (4.3 MB ZIP). [file pbio.0020422.st001.zip › NielsenEtAl/html/702.html]

AN5497.1.NCU05983.1.MG01049.1.FG08452.1


```
 CLUSTAL W (1.82) Multiple Sequence Alignments - Introns Inserted


Sequence 1: NCU05983.1	236 aa
Sequence 2: MG01049.1	233 aa
Sequence 3: FG08452.1	235 aa
Sequence 4: AN5497.1	225 aa
Alignment Length: 241 aa
Number Identitical Residues: 104 aa
Alignment Score (without introns) 5609


MG01049.1 	MPSTDS--IPKSNFDAIPDVIQAFK1NGEFVVVLDDPSRENEADLIIAAESVTTEQMAFM
NCU05983.1	MASQQESRMDPSAFDSIPDTVEAFR1NGEFVVVLDDPGRENEADLIIAAESVTAQQMAFM
FG08452.1 	MPAT----IPQNNFDSIPDSIEAFR1NGEFLVVLDDPSRENEADLIIAAEDVTTEKMGFL
AN5497.1  	MPSPTD---PALQFDSIEDTIKAFS1NGEFIVVLDSQDRENEGDLIIAAESITDAQMAFL
          	*.:  .       **:* * ::**  ****:****. .****.*******.:*  :*.*:

MG01049.1 	VRHSS~GLICAPLTPERTTALDLPQMVTHNADPRGTAYTVSVDAEHPSTTTGISAHDRAL
NCU05983.1	VRYSS~GLICAPILPQRTLDLDLPQMVGHNEDPRATAYTISVDAADPSVTTGISAHDRAL
FG08452.1 	IRHSS~GYVCVPLSPELTKSLDLPQMVPNNQDPRGTAYTISVDSADPSVTTGISATDRAL
AN5497.1  	VRYTS2GLICAPITPEIAERLQLPQMVLENTDPKGTAYTVSIDSADPSITTGISAHDRAL
          	:*::* * :*.*: *: :  *:***** .* **:.****:*:*: .** ****** ****

MG01049.1 	ACRMLAAPDAQPSHFRRPGHVFPLRAVAGGVRARRGHTEAGVELCRLAGKRPVAVISEIV
NCU05983.1	VCRVLADEKTKPADLRRPGHVFPLRARPGGVRQRRGHTEAAIDLCRMAGKKPAAVISELV
FG08452.1 	ACRTLADPKARPDSFRRPGHVLPLRARPGGVRQRPGHTEAATEFCRLAGKAQAAAICEII
AN5497.1  	ACRTLASPSARPQDFRRPGHIIPLQAKAGGVRQRKGHTEAAVEFCRLAGKAPAGVIAELV
          	.** **  .::*  :*****::**:* .**** * *****. ::**:***  ...*.*::

MG01049.1 	DDGQEVEG-RAVRAAPGMLRGDECVAFARRWGLK~VCT~IEDMIAHVEKTEGKL-ETNGS
NCU05983.1	DDGREVEG-QAVREEPGMMRGEQCVAFARRWGLK~VCT~IEDMVKYLEEKEGKLGELNGS
FG08452.1 	DDGEETPG-QALVQNHGMLRGEACITFARKWGLK~VCT~IADLVAYVEKTEGKL-ESNGA
AN5497.1  	EDGEVVEGVAEIRGNNGMMRRDGCLKFGKKWGLK0LYD1RN--IAYYILINWFI------
          	:**. . *   :    **:* : *: *.::**** :       : :    :  :      

MG01049.1 	G----
NCU05983.1	S----
FG08452.1 	ETEGN
AN5497.1  	-----
          	
```
